# Major breakthroughs in hand surgery

**DOI:** 10.1186/1753-6561-9-S3-A6

**Published:** 2015-05-19

**Authors:** SP Chow

**Affiliations:** 1 Department of Orthopaedics and Traumatology, University of Hong Kong, Hong Kong

## 

During my 40 years of involvement with hand surgery, I have witnessed the following major breakthroughs:

- Tendon surgery and tendon program

- Development of artificial finger joints

- Microsurgery

- Various fixation systems for fractures

- Wrist arthroscopy

Certain breakthroughs are appearing in the horizon:

- 3-D printing

- Tissue engineering

- Genetic engineering

- Intra-uterine surgery

At organizational level, the following are also important:

- Team approach for rehabilitation

- IFSSH, APFSSH, national societies

However, all these technical and scientific advances should be guided by future breakthroughs in the arts and humanities of medicine – the love and care for our patients.

